# Analysis of tigecycline resistance development in clinical *Acinetobacter baumannii* isolates through a combined genomic and transcriptomic approach

**DOI:** 10.1038/srep26930

**Published:** 2016-05-31

**Authors:** Lin Liu, Yujun Cui, Beiwen Zheng, Saiping Jiang, Wei Yu, Ping Shen, Jinru Ji, Lanjuan Li, Nan Qin, Yonghong Xiao

**Affiliations:** 1 State Key Laboratory for Diagnosis and Treatment of Infectious Disease, The First Affiliated Hospital, College of Medicine, Zhejiang University, 310003 Hangzhou, China; 2 Collaborative Innovation Center for Diagnosis and Treatment of Infectious Diseases, Zhejiang University, 310003 Hangzhou, China; 3 State Key Laboratory of Pathogen and Biosecurity, Beijing Institute of Microbiology and Epidemiology, Beijing, China

## Abstract

Tigecycline (Tgc) is considered a last-resort antibiotic for the treatment of multi-drug resistant bacteria. To study Tgc resistance development in the important nosocomial pathogen *Acinetobacter baumannii*, we adopted six clinical isolates from three patients undergoing antibiotic treatment, and bacterial genomic sequences and seven strand-specific transcriptomes were studied. Interestingly, the Tgc-intermediate 2015ZJAB1 only differed from Tgc-resistant 2015ZJAB2 in an SNP-clustered region including OprD, a sugar-type MFS permease, and a LuxR-type transcriptional regulator. Surprisingly, an almost identical region was found in 2015ZJAB3, which supports the possibility of a homologous recombination event that increased Tgc resistance. Furthermore, comparative transcriptomic analysis identified significantly regulated genes associated with Tgc resistance, which was verified using qRT-PCR. Three enriched COG categories included amino acid transport and metabolism, transcription, and inorganic ion transport and metabolism. KEGG analysis revealed common features under Tgc conditions, including up regulated benzoate degradation and a less active TCA cycle. This may be related to selective antimicrobial pressure in the environment and adaptation by lowering metabolism. This study provides the first report of an *in vivo* evolutionary process that included a putative homologous recombination event conferring Tgc resistance in clinical *A. baumannii* isolates in which transcriptome analysis revealed resistance-conferring genes and related metabolism characteristics.

*Acinetobacter baumannii*, a Gram-negative opportunistic pathogen in humans, is becoming increasingly important as a nosocomial pathogen. It is commonly found in infections of the lung, blood stream, urinary tract and wounds in intensive care units, where it especially affects people with immune deficiency^1^. Owing to the advantages of its ability to rapidly acquire resistance genes such as *tetA, tetB* and *tet*X, and to survive exposure to surface microcides, multidrug-resistant (MDR) *A. baumannii* is becoming a severe threat and compromising healthcare outcomes worldwide^2^. Risk factors for *A. baumannii* infections include antibiotic pre-treatment, surgery as well as the use of ventilators and other medical instruments. Carbapenems (i.e., imipenem, meropenem) are recommended agents for the treatment of MDR *A. baumannii* infections. However, a significant rise in carbapenem-resistant *A. baumannii* has been reported globally^3^. FDA-approved Tgc, the first identified glycylcycline antibiotic, which mimics the structure of tetracycline but is effective against tetracycline resistant bacteria^4^, has been recommended for the treatment of complicated intra-abdominal infections, skin infections, and community-acquired pneumoniae since 2007. Hospitals in China began to use Tgc in late 2012. Tgc was recognized as a “last-resort” antibiotic because of its bacteriostatic activity against MDR *Acinetobacter.* It acts as a protein synthesis inhibitor by binding to the 30S ribosomal subunit of bacteria and thereby blocking entry of aminoacyl-tRNA into the A site of the ribosome during prokaryotic translation^5^. Since its first use in the clinic, an increasing number of medical cases have been reported concerning the development of bacterial Tgc resistance (Tgc-R) and the associated clinical outcome^6^. Some studies have used molecular typing to elucidate the epidemic features of Tgc-R *A. baumannii*. With molecular-based detection, some researchers have investigated the prevalence of certain resistance-determinant genes, such as Nowark’s work that studied the prevalence of resistance nodulation division (RND)-type efflux pumps among 144 epidemiological *A. baumannii* isolates from locations around the world^7^.

Recent publications have indicated that the phenotype of antibiotic resistance (AR) is related to a concerted activity of antibiotic resistant genes and other genetic elements, such as basic metabolic components. These elements together organize into a “resistome”^8^. Like AR genes, they can be intrinsic or acquired. Resistome research is developing quickly with the aid of next-generation sequencing and “omics” applications. Using genomics and transcriptomics techniques, the profiles of not only a few AR genes but also the overall landscape and interactions of the genetic network can be characterized. To date, the Tgc resistance mechanism has been mainly investigated in *Klebsiella pneumonia, E. coli,* and *A. baumannii*. It has been reported that the Tgc-resistance mechanism is mainly mediated by efflux and regulators, particularly RND-type efflux pumps encoded by *adeB*, *adeJ* and *ade*^9^. In addition, global regulators involved in the SOS responsive system, adaption or virulence-related co-evolution may also contribute to increased Tgc resistance. Cyclic-di-GMP is a secondary messenger involved in bacterial adhesive ability and extracellular polysaccharide (EPS) biogenesis, and it may facilitate resistance through biofilm barrier formation or through altered metabolism within the biofilm^7^,^10^,^11^. Other related AR genes include those that encode metabolic enzymes, such as methyltransferase, acyltransferase and hydrogenase^12^, and some of their functions have been studied in wet lab experiments.

Despite the numerous case reports on Tgc-R *A. baumannii* infections and epidemiological studies using molecular typing, little is known about the development of Tgc resistance in clinical *A. baumannii* strains, or about the contributions of genomic mutations or transcriptional regulation. The design of the present study involved the selection clinical *A. baumannii* isolates from the same patient during antibiotic treatment. Subsequently, through comparative genomic and transcriptomic analyses, we identified genome-wide variations and differentially expressed genes that are responsible for the increased capacity for Tgc resistance that develops during antibiotic treatment. Finally, GO, COG and KEGG analyses were carried out to identify bacterial functional and metabolism characteristics related to AR capacities.

## Results

### Strain groups, AR profiles and clinical records

This project adopted *A. baumannii* strains isolated from the same patient during antibiotic treatment with the same multilocus sequence type (MLST) and different Tgc resistance levels. As a result, there were a total of six strains from three patients. Patient I provided strains 2015ZJAB1, 2015ZJAB2 and 2015ZJAB3, which belonged to ST451, and all were isolated from sputum samples; 2015ZJAB4, which was isolated from a bile sample of patient II, belonged to ST195; 2015ZJAB7 and 2015ZJAB8, which were isolated from drain fluid of patient III, belonged to ST208. The isolation time of the six strains and antibiotics administered to the patients are shown in Figure S1. Briefly, for Patient I, Tgc was administered two days after the isolation of 2015ZJAB1, and 2015ZJAB2 was isolated on the second day of the second treatment course of Tgc, i.e., 7 days after the first treatment course and 5 days after the interval period. 2015ZJAB3 was isolated three weeks after the end of Tgc treatment. For Patient II, Tgc was administered 7 days after the isolation of 2015ZJAB4. For Patient III, Tgc was not administered in the clinical treatment.

The AR spectrum of the six strains was tested against major clinical antibiotics, and the minimal inhibition concentration (MIC) of Tgc and other antibiotics was determined using broth micro-dilution methods in fresh Mueller-Hinton broth medium. All of the strains were resistant to multiple major clinical antibiotics (Table 1). Strain 2015ZJAB2 was resistant to all major clinical antibiotics tested, including amikacin, cefoperazone, and Tgc, whereas 2015ZJAB3 showed reduced resistance to Tgc and piperacillin/tazobactam. 2015ZJAB1 was additionally intermediately resistant to imipenem, aztreonam and Tgc.

### Comparative genomic analysis

#### Sequencing and assembled data

The general features of the six *A. baumannii* genomes investigated in this study are shown in Table 2. An average of 671 Mbp of high-quality data were generated for each of the six strains, corresponding to an average sequencing depth of 163-fold. CDSs were predicted in the *A. baumannii* genomes with an average and minimum length of 920 bp and 911 bp, respectively.

#### SNP, InDel and homologous recombination

As expected, strains within each group (patient) possessed significant genomic sequence homology, a finding that was consistent with the clinical information of a relatively short isolation interval time and common strain type (ST). SNP and InDels were analyzed within each group.

**Figure 1:**
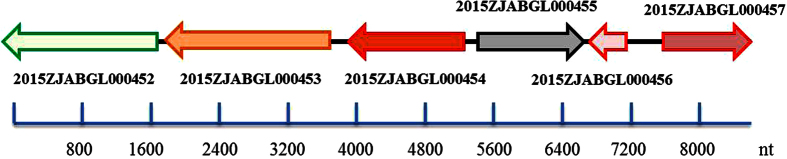
Hot single nucleotide polymorphism (SNP) mutation region involving six adjacent genes. The nucleotide length marker is shown below.

In Group 1 (Patient I), the earliest isolated strain Tgc-intermediate (Tgc-I) 2015ZJAB1 was selected as the inner-group reference for SNP comparison with 2015ZJAB2 and 2015ZJAB3. In total, 3,255 SNPs were shared between 2015ZJAB3 and the other two strains (Table S1). However, 319 SNPs were observed between 2015ZJAB1 and 2015ZJAB2, and over 95% of them were clustered in a region including six adjacent genes (Fig. 1). Further analysis identified no IS elements in or around this region. Interestingly, an almost identical region to the SNP-clustered fragment was identified in 2015ZJAB3, implying the occurrence of a possible genomic shift between 2015ZJAB1 and 2015ZJAB3, resulting in 2015ZJAB2 which displayed an increased capacity for Tgc resistance.

Among the six genes with high-density SNPs, two genes were located on the positive strand (Fig. 1); one was the LuxR family transcriptional regulator harboring two premature stop mutations (g2015ZJAB1L000455), and the other was a membrane protein (g2015ZJAB1L000457). Of the four other genes on the negative strand, three were located adjacently. The OprD outer membrane porin (g2015ZJAB1L000452; involved in transport of antibiotics into cells) was upstream of a sugar-transporter-like major facilitator superfamily (MFS) permease (g2015ZJAB1L000453; involved in amino acid transport and metabolism), which preceded the dihydroidipicolinate synthase/N-acetylneuraminate lyase (g2015ZJAB1L000454; involved in cell envelope biogenesis). A small hypothetical protein-coding gene (g2015ZJAB1L000456) was located upstream of these three genes. The intergenic mutations, as well as 7 other SNPs located outside of the region, were not found to contain obvious regulator-binding sites or to affect any other functions.

Blast analysis with CDD (conserved domain database) showed that some nonsynonymous mutations were located in the conserved motifs of g2015ZJAB1L000453, g2015ZJAB1L000452 and g2015ZJAB1L000455, which could influence the coded proteins’ functions.

Minor SNP mutations were found in Group 3 (Patient III) strains. Notably, two SNPs in gene g2015ZJAB7GL001469 encoding Glycerol-3-phosphate dehydrogenase (GPDH) were identified. Because it plays an important role in carbohydrate and lipid metabolism, it might be indirectly related to AR.

Only one InDel mutation was identified in Group 1 (Patient I) strains. Genes g2015ZJAB1GL001624 and g2015ZJAB2GL001537 had a 39-bp deletion compared with g2015ZJAB3GL001828. It was annotated as a cation diffusion facilitator protein. No InDels were found in Group 3 (Patient III) strains.

#### Co-linearity analysis

**Figure 2:**
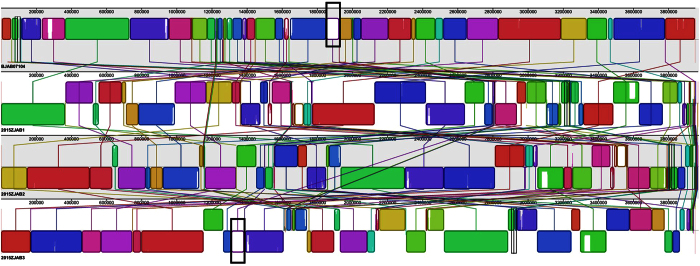
Co-linearity analysis of 2015ZJAB1, 2015ZJAB2, 2015ZJAB3 with BJAB07104. The ~80-K region with the black frame was present in the reference genome BJAB07104 and 2015ZJAB3 but absent in 2015ZJAB1 and 2015ZJAB2.

In addition to many small nucleotide differences, co-linearity analysis can reveal large structural variations. We identified an ~80-kb chromosomal region (1850794-1924731 in reference genome BJAB07104). While present in 2015ZJAB3, it was, however, absent in 2015ZJAB1 and 2015ZJAB2 (Fig. 2). The region comprised 60 genes, including several transcriptional regulators, membrane proteins and transporters (Table S2). The loss of a relatively large region with many annotated genes, including some AR genes, may alter some aspects of the bacterial phenotype. *A. baumannii* ATCC17978 shared the same 80 K region with 2015ZJAB3. With an identity of approximately 80%, epidemic MDR strains ZW85-1, D36, BJAB0715, XH386, MDR-TJ, and MDR-ZJ06 also retained this region.

A small plasmid highly homologous to plasmid NC_02178 and present in BJAB07104 was also identified in 2015ZJAB1. In these aligned plasmid contigs, resistance genes *armA*, *msrE*, *sull* and *catB8* were annotated. However, the larger plasmid present in BJAB07104 was absent in all patient I strains. The sequencing depth of the plasmid was 240-fold, more than the average genomic region depth of 161-fold. Efficient isolation of both chromosomal and plasmid DNA was obtained using the QiaAmp DNA extraction kit.

**Figure 3:**
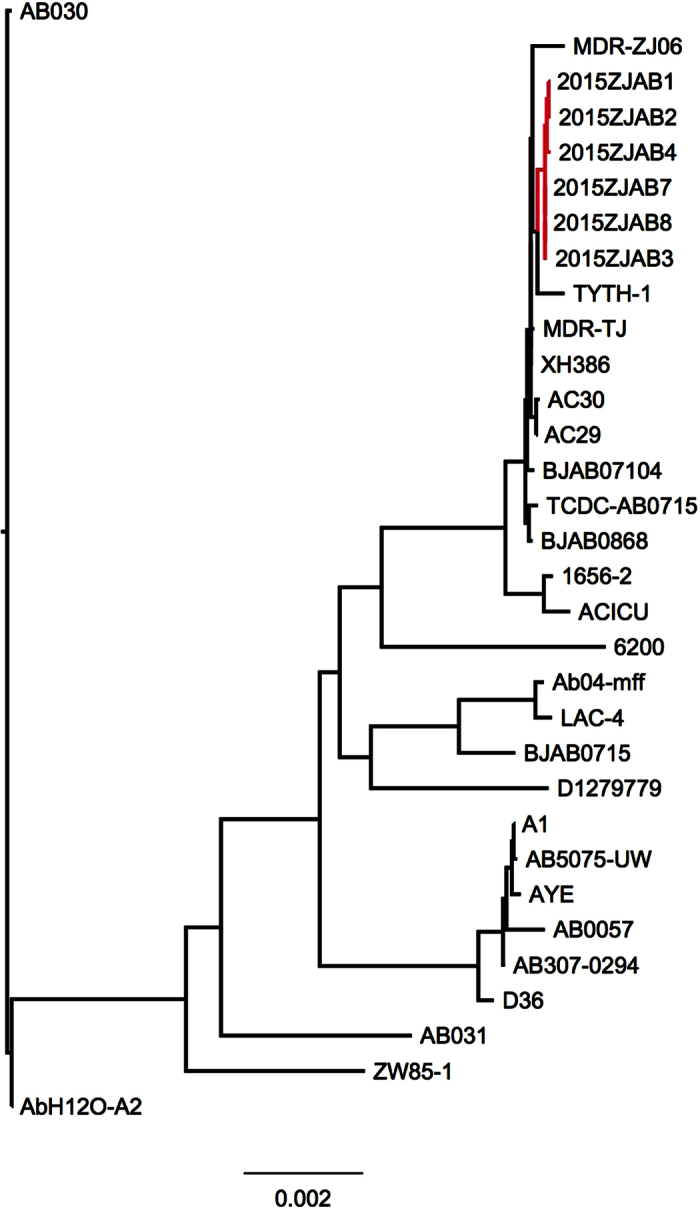
Phylogenetic tree of six genomes involved in this study based on core gene SNPs.

Despite the different strain types (STs) and structural variations, all six *A. baumannii* strains in this report were phylogenetically close based on core gene SNPs (Fig. 3). They were rooted close to MDR-ZJ01, MDR-TJ, and BJAB07104. Most of these strains were isolated in geographically close locations in eastern China.

#### Genomic islands

Fourteen islands were predicted in strain 2015ZJAB1 (http://www.pathogenomics.sfu.ca). AR genes were annotated by both ARDB (http://ardb.cbcb.umd.edu) and CARD (http://arpcard.mcmaster.ca/). The latter database was relatively new and generated more annotated AR genes and classifications than the traditional ARDB database. Most of the additionally annotated AR genes belong to the *ade* family. After gene annotation with ARDB, CARD, COG, KEGG and VFDB databases, we found two 26-kb islands (217314 and 217303) containing four different AR genes (g2015ZJAB1GL000172, 000174, 000170 and 001319) that coded for beta-lactam, sulfonamide, chloramphenicol and aminoglycoside resistance, respectively. In strain 2015ZJAB2, the annotation results were similar, while gene g2015ZJAB2GL000139 had two new annotations using the CARD database. In strain 2015ZJAB3, four different AR genes (g2015ZJAB3GL000161, g2015ZJAB3GL001496, g2015ZJAB3GL05 and g2015ZJAB3GL000162) were annotated for beta-lactam and aminoglycoside resistance using ARDB; however, when using CARD, they were annotated with more details. By comparison, the resistant strain 2015ZJAB2 contained two more AR genes coding for sulfonamide and chloramphenicol resistance.

In strain 2015ZJAB4, an island was found on scaffold 17 that contained two antibiotic genes (g2015ZJAB4GL001778, 001779) coding for proteins that modify aminoglycosides through phosphorylation. In 2015ZJAB7, three different AR genes (g2015ZJAB7GL000395, 000396 and 000397) were annotated on two islands (4136526 and 4148731) with the ARDB and CARD database, and g2015ZJAB7GL000400 was additionally annotated using CARD. In strain 2015ZJAB8, 16 islands appeared in the genome, but no resistance genes were annotated with either database.

#### Comparative analysis of integrin/integrase in *A. baumannii*

After the genome blast with the ISfinder database, IS sequences were identified based on sequence homology and coverage mapped, whereby we found that most of the IS lengths were approximately 1 kb (Table S3). Strains 2015ZJAB1 and 2015ZJAB2 shared the same targeted IS elements, which were greater in number than in 2015ZJAB3. Compared with 2015ZJAB3, 2015ZJAB1 and 2015ZJAB2 did not possess ISAba19, 2, or 18, but had ISAba22, and ISEc29, 28 and 35. The integron was assessed for *attC* sequences using rpsblast against CDD databases, and no integrase was found.

#### Comparative transcriptomics

Seven strand-specific transcriptome profiles of three strains 2015ZJAB1, 2015ZJAB2 and 2015ZJAB3 under three Tgc conditions (0, 0.5 and 1 mg/L) were characterized and comparatively analyzed. One transcriptome of 2015ZJAB3 without Tgc was included in this study, due to prolonged incubation time with Tgc supplement over 40 hours.

**Figure 4:**
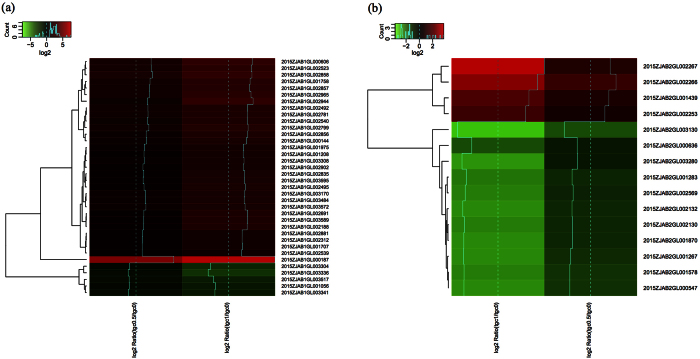
Heatmap of significantly differentially expressed genes in 2015ZJAB1 and 2015ZJAB2 with a gradient increase in the tigecycline concentration. The colors of the heatmap are log2 ratios of differential expression, with green representing down-regulation, and red representing up-regulation. The blue line represents the log2 ratio value of each gene, and the blue dash line means 0. The resulting tree figures were displayed using heamp.2 in R software.

In 2015ZJAB1, when Tgc concentrations were increased from 0 to 0.5 and from 0.5 to 1 mg/L, 63 genes were identified as being continuously up regulated over 2-fold at each increase in Tgc concentration (Fig. 4, Table S4). These genes were considered as “significantly up-regulated genes”. Among them, 39 genes were up regulated more than 8-fold. Half of the significant genes were annotated using the current databases, including 11 genes coding for efflux pumps, permeases and outer membrane proteins involved in carbohydrate, lipid, nucleotide, amino acid and inorganic ion transport and metabolism; 6 genes involved in cell envelope (cell wall and membrane) biogenesis; 6 genes involved in the transcription and translation of active proteins including 4 transcription regulators and binding proteins; 2 genes coding for proteins involved in secondary metabolite biogenesis, transport and catabolism; 1 gene coding for an integrase involved in replication, recombination and repair; and 1 gene involved in signal transduction. Another 9 proteins were assigned to prediction only or unknown function. By contrast, only 6 genes were “significantly down-regulated” with increasing Tgc concentration, 2 of which were annotated as being involved in cell envelope (cell wall and membrane) biogenesis and ribosomal structure and biogenesis (Table S4).

In 2015ZJAB2, 11 genes were significantly up-regulated with increasing Tgc concentration (Fig. 4), and only one gene was annotated using KEGG (K07345) as being involved in cell motility, intracellular trafficking, secretion and vesicular transport. The transcription of 16 genes significantly decreased, and 2 genes were annotated as taurine transport system permease proteins (Table S5). Comparatively, 2015ZJAB2 exhibited fewer up-regulated genes than 2015ZJAB1 but contained more down-regulated genes. The transcriptome profile of 2015ZJAB3 was distinct from 2015ZJAB1 and 2015ZJAB2, reflected in different GO term patterns, and it was taken from the culture without Tgc.

The transcriptional levels of SNPs or InDel mutation-harbored genes in 2015ZJAB1 and 2015ZJAB2 were comparatively analyzed. When Tgc was added and increased from 0 to 0.5 and from 0.5 to 1 mg/L, the MFS permease expression increased by 2.68- and 3.87-fold in Tgc-I 2015ZJAB1, respectively, while the homologous gene expression in the Tgc-R strain 2015ZJAB2 varied only slightly (within 20%). The OprD outer membrane porin varied similarly and increased by 2.27- and 2.72-fold in 2015ZJAB1, respectively, with each increased concentration of Tgc, while the expression in 2015ZJAB2 varied within 10% with each increase in Tgc concentration. However, for the LuxR-type transcriptional regulator, its expression was gradually up regulated up to 2.72-fold in the Tgc-I strain, while the stop-codon-harboring homologous gene in the Tgc-R strain was slightly up-regulated at Tgc0.5 and then expression decreased dramatically at Tgc1. Theoretically, stop codon mutations do not affect the transcription level of a gene; hence, further investigation is needed to explain this result. The hypothetical protein was notably up-regulated at both Tgc concentrations in both strains. Interestingly, the annotated membrane protein g2015ZJAB1GL000457 was slightly continuously up-regulated in the Tgc-I strain but obviously down-regulated in the Tgc-R strain, especially with 1 mg/L of Tgc. Insignificant differences in the expression of the InDel mutation genes were observed in 2015ZJAB1 and 2015ZJAB2.

Generally, mutation-containing genes exhibited greater regulation of expression in 2015ZJAB1 than in 2015ZJAB2. In 2015ZJAB1, up-regulation of two- to three-fold was observed; however, 2015ZJAB2 exhibited a maximum variation in expression of 30% or was slightly down-regulated. Considering the many genes for which expression changed by over eight fold (Fig. 4), we suggest that not only mutated genes but also transcriptional regulation contributed to the altered AR of the bacteria.

#### qRT-PCR

Thirty-four significantly differentially expressed and mutation-harboring genes were analyzed using qRT-PCR. RNA was isolated from log-phase cultures of 2015ZJAB1 and 2015ZJAB2 at Tgc concentrations of 0, 0.5 and 1 mg/L. All of the genes and primers used are listed in Table S6. Genes being tested in qRT-PCR include significantly up-regulated and down-regulated AR genes in 2015ZJAB1 and 2015ZJAB2 revealed by comparative transcriptomic analyses, SNP and InDel mutation genes, as well as some genes that are dramatically differentially expressed over 32-fold with increase of Tgc concentraion, but only varied slightly or even reversely regulated under higher Tgc pressure. The comparison between the transcriptome and qPCR data is shown in Table S7. Generally, qPCR data exhibited the same gene variation trend as the transcriptome profile but to a lesser extent.

### Functional analysis

#### GO analysis and COG classification

GO analysis revealed distinct differently expressed gene enrichment profiles in Tgc-I 2015ZJAB1 and Tgc-R 2015ZJAB2 (Table S8). In the Tgc-I strain, differentially expressed genes were enriched in terms of DNA and nucleic acid binding, biological regulation and regulation of biological processes, and regulation of cellular processes (Table S8). The most dramatic increase in gene transcription occurred between the Tgc concentrations of 0 and 1 mg/L. By contrast, down-regulation gene patterns were the most dramatically different between the Tgc concentrations of 0.5 and 1 mg/L, with GO terms enriched with respect to membranes in cellular components, oxidoreductase activity as well as transporter activity for molecular functions. Orthologous genes were classified into COG groups. The top five classifications with notable differences were “general prediction only”, “amino acid transport and metabolism”, “transcription”, “unknown function” and “inorganic ion transport and metabolism”.

#### KEGG analysis

**Figure 5:**
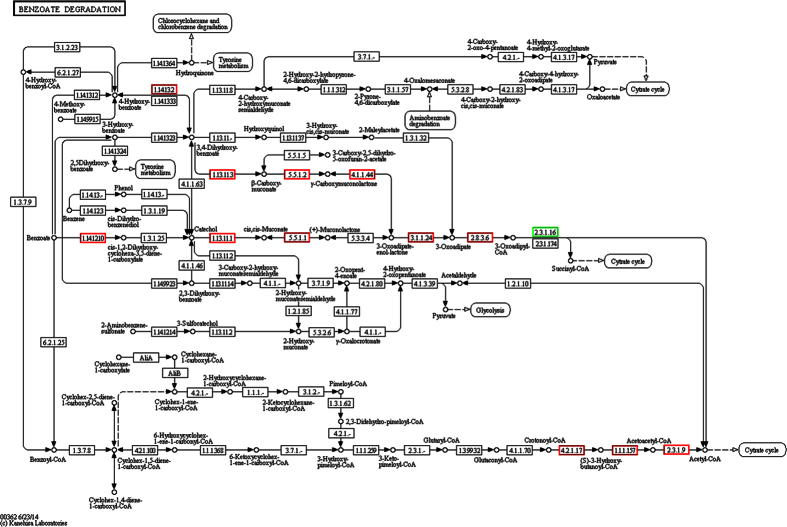
Common significantly up-regulated genes involved in the benzoate degradation pathway in both 2015ZJAB1 and 2015ZJAB2. The significantly up-regulated genes are shown in red frames in 2015ZJAB1. In 2015ZJAB2, most of these genes were also significantly up regulated except one gene, 1.13.11.1, whose expression was not significantly different, and the three red-framed genes at the bottom were down regulated.

**Figure 6:**
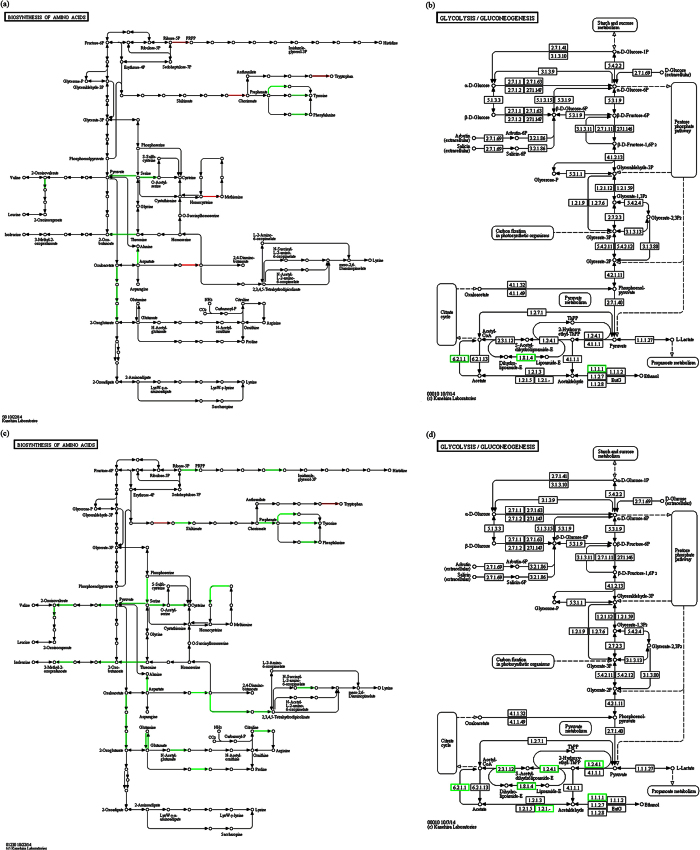
Less active metabolism of Tgc-R 2015ZJAB2. Under tigecycline conditions of 0.5 mg/L (**a**,**b**) and 1 mg/L (**c**,**d**), compared with Tgc-I 2015ZJAB1, the Tgc-R strain 2015ZJAB2 showed less active metabolism in the glycolysis pathway (**b**,**d**) and amino acid biosynthesis pathway (**a**,**c**). Green indicates significantly reduced transcription, and red represents significantly increased transcription.

KEGG analysis revealed the accumulation of differentially expressed genes in certain pathways. Benzoate degradation was continuously up regulated in both 2015ZJAB1 and 2015ZJAB2 with each increase in Tgc concentration, while the TCA cycle was the common continuously down-regulated pathway in the presence of Tgc. As shown in Fig. 5, red-framed genes involved in benzoate degradation repeatedly appeared in both bacterial isolates as significantly up-regulated genes. Figure 6 shows reduced metabolism represented by the TCA cycle and amino acid biogenesis in 2015ZJAB2.

#### AR gene annotation

AR genes were annotated using ARDB and CARD. In 2015ZJAB1, 29 AR genes were annotated using ARDB, and 49 AR genes were annotated using CARD, of which 14 belonged to the *ade* family. The same result was observed in 2015ZJAB2. In 2015ZJAB3, 27 AR genes were annotated using ARDB, and 46 AR genes were annotated using CARD, of which 15 belonged to the *ade* family. A comparison of ARDB-annotated AR genes in three patient I strains revealed that several resistance genes, i.e., for streptomycin (spectinomycin), ribostamycin (lividomycin, gentamicin_b, paromomycin, kanamycin, neomycin) and aminoglycoside (fluoramphenicol), were only found in 2015ZJAB1 and 2015ZJAB2. On the other hand, the resistance gene for paromomycin (lividomycin, neomycin, gentamicin_b, kanamycin, and ribostamycin) was only found in 2015ZJAB3. CARD annotation analysis revealed that four resistance genes (*sull*, *msrE*, *catB8* and *armA*) were only found in 2015ZJAB1 and 2015ZJAB2.

#### Virulence annotation by the VFDB database

Vfdb annotation revealed that strain 2015ZJAB3 had the most annotated Vfs (168 genes), followed by 2015ZJAB2 (157 genes) and 2015ZJAB1 (155 genes). In 2015ZJAB3, K12056–K12068 and K12072, which represented the conjugal transfer mating pair stabilization protein, conjugal transfer pilus assembly protein, conjugal transfer ATP-binding protein and conjugal transfer pilin signal peptidase, were not present. When combined with transcriptomic analysis, there were two virulence genes g2015ZJAB1GL002857 (a multidrug efflux pump for cations) and g2015ZJAB1GL002858 (a membrane-fusion protein) that were found to be significantly up regulated and one virulence factor g2015ZJAB1GL001056 (outer membrane and peptidoglycan-associated lipo-protein) that was significantly down regulated. By contrast, 2015ZJAB2 did not exhibit the significantly up regulated virulence factors but significantly down regulated the homologous virulence gene g2015ZJAB2GL001905.

## Discussion

Nosocomial pathogen multidrug-resistant (MDR) *A. baumannii* has become a severe threat and is compromising healthcare outcomes worldwide^2^. It is becoming resistant to Tgc, a “last-resort” antibiotic. Comparative genomic and transcriptomic analyses can provide a multi-level analysis of resistance mechanisms^13^,^14^. To investigate Tgc resistance development and mechanisms in clinical MDR *A. baumannii*, we selected *A. baumannii* strains isolated from the same patient during antibiotic treatment. Through genome sequencing and comparative analysis, genomic mutations were identified that corresponded with phenotypic differences in MDR strains. The most interesting findings were detected in patient I strains (2015ZJAB1, 2015ZJAB2 and 2015ZJAB3), which were named based on the order of isolation. They belonged to ST451, a relatively rare type, which also belongs to the global clone GC2 and clone complex CC92. Compared with Tgc-I 2015ZJAB1, mutations in strain 2015ZJAB2 were revealed to be surprisingly intensively clustered in one region that harbored six genes, including an OprD-like outer membrane porin, a LuxR-type transcriptional regulator and a sugar-type MFS membrane protein. An almost identical region was detected in 2015ZJAB3, implying the occurrence of a possible recombination event that conferred increased Tgc resistance. Clinical antibiotic treatment could provide natural selection power that is discussed later in this article. There is no IS element adjacent to this region or involvement of a genomic island. To our knowledge, this study is the first report of Tgc resistance evolution in clinical *A. baumannii* isolates via putative homologous recombination. It has been reported that a clinical *A. baumannii* natural competent state and relevant gene expression were related to the growth rate and were affected by DNA-toxic antimicrobials^15^. However, the mechanism underlying the homologous recombination event remains unknown.

The six mutation-harboring genes associated with increased Tgc resistance deserve further discussion, particularly those with nonsynonymous mutations located in conserved regions that could affect the coded protein’s function. The gene g2015ZJAB1GL000452 was annotated as an OprD-like outer membrane porin based on computerized annotation and homology analysis using CDD. It has been reported that Tgc resistance can be mediated by active functions of efflux pumps and regulators such as *adeG, adeJ and adeB*^9^. Regarding outer membranes, an OprD homolog was identified in *A. baumannii* as associated with the influx of amino acids and antibiotics such as imipenem^16^. Another study showed that, under tetracycline stress, major outer membrane proteins were actively regulated to significantly decrease expression and over-secretion, but this outcome was not corroborated on the transcriptional level^17^. Most recently, it was revealed that its function in the presence of carbapenem might involve adaptation of the bacterium to magnesium- or iron-depleted conditions^18^. The comparative transcriptomic data showed that 2.27- and 2.72-fold up regulation of this OprD-like protein was observed in Tgc-I with increasing Tgc concentrations, while in Tgc-R only minor up regulation was detected. Taken together, the role of OprD in Tgc resistance might not be direct and needs further study.

Another gene, g2015ZJAB1GL000455, was identified as a putative LuxR-type transcriptional regulator. It was originally identified and investigated in *Vibrio fischeri* in studies on quorum sensing. Now, it has been identified to be involved in various biological processes. In *A. baumannii*, it is involved in the regulation of quorum sensing as well as in metabolism regulation and bacterial virulence^19^. Cerqueira *et al.* identified that the LuxR_C-like gene *gacA* functions by interacting with GacS and is involved in the phenylacetic acid catabolic pathway and virulence of the bacterium^19^. It was up regulated up to 2.72-fold with increase of Tgc in the Tgc-I strain, but only minor up regulation was observed in Tgc-R at the mild Tgc condition, with a dramatic down regulation under higher Tgc pressure. Mutations in the putative LuxR-type transcriptional regulator may affect Tgc resistance via the regulation of its target genes that remain to be determined. The gene g2015ZJAB1GL000453 was annotated as a sugar-type MFS permease. In *A. baumannii*, MFS efflux-pump-mediated resistance to antibiotics is commonly observed^20^. This type of efflux pump is known to be involved in carbohydrate metabolism, amino acid transport and metabolism, inorganic ion and drug transport and is related to the resistance to structurally different drugs such as chloramphenicol, erythromycin and tetracycline. Although the efflux determinants *tetA* and *tetB* confer tetracycline resistance, they do not affect glycylcyclines such as Tgc. However, it was reported that with the presence of tigecycline, MFS pumps were up regulated in *A. baumannii*^14^. Upon closer inspection, the gene belongs to the sugar porter (SP) subgroup of the MFS superfamily (Transporter classification database, http://www.tcdb.org) and is mainly involved in carbohydrate transport and metabolism. Subsequent transcriptome data showed that several SP-type MFS permeases were significantly up regulated in Tgc-I 2015ZJAB1, indicating the role of this permease in Tgc resistance. However, the transcription of the homologous gene was maintained with minor variation in Tgc-R 2015ZJAB2. Notably, it is involved in benzoate transport, and benzoate degradation was commonly up regulated in both Tgc-I and Tgc-R strains under all Tgc concentrations. However, the association of benzoate degradation with altered benzoate transport cannot be ruled out, and it may possibly contribute to the ability of the bacterium to adapt to the presence of microcide.

The mutation-driven pressure was probably due to the use of antibiotics during clinical treatment. Based on the analysis of clinical records (Figure S1), only 2015ZJAB2 was isolated during the Tgc treatment period, which was approximately two weeks after 2015ZJAB1 and three weeks before 2015ZJAB3. The time interval between the isolation of different strains and antibiotic treatment might not be sufficient for the occurrence of novel mutations changing the resistance phenotype. This finding is in accordance with the Ka/Ks analysis result (<1, data not shown). However, it is plausible for some mutations, such as homologous recombination or an acquisition event, to have occurred and affected AR. The clinical records also showed that Tgc insusceptibility could appear before (patient II) or without (patient III) Tgc administration (Figure S1); thus, Tgc might not be a prerequisite for the development of Tgc resistance. Instead, it could be induced by antibiotics such as a cefalo-type and carbapenem, which were administered to patients II and III. Resistance to cefoperazone and imipenem involves regulated activities in efflux pumps and porins^21^ that may also facilitate resistance to Tgc.

Transcriptomic and qPCR data showed limited variations in the transcription of the six mutated genes under Tgc conditions. The entire transcriptomic profiles of the Tgc-I and Tgc-R strains during Tgc treatment were analyzed to provide insights into gene regulation and adaption variations associated with AR. Tgc concentrations of 0, 0.5 (mild stress) and 1 mg/L (stringent stress)^22^ were used to identify significantly regulated genes, and a dynamic change of cells and transcriptional response process was revealed. Genes were characterized in this study as those that were consistently up or down regulated under each Tgc concentration, with a more than two-fold change at each step and a final variation that was greater than four-fold. Most of the significantly differentially expressed genes were involved in membrane transport and cell envelope biogenesis (Tables S4 and S5). There were 40 genes for which expression varied over eight-fold among 64 significant genes. Among these genes, not only AR genes but also transcriptional regulators and metabolic genes were identified, indicating a concerted action of “resistome” factors associated with AR^14^. The complexly and significantly regulated genes identified in this study need to be investigated further to fully characterize their roles in the resistome network.

In the analysis of significantly regulated genes and their involvement in the accumulated functions and metabolic pathways using the GO and KEGG databases, it is interesting to note that both strains under Tgc conditions shared an up-regulated benzoate degradation pathway (Fig. 5) and a down-regulated TCA cycle pathway (Fig. 6). Benzoate is normally used as an antimicrobial agent in food preservation or cleaning products. The accumulation and up-regulation of benzoate degradation may reflect *A. baumannii*’s capacity to survive in an environment with benzoate, thus facilitating the existence of the bacterium as an opportunistic pathogen in hospitals. TCA de-activation may favor its adaption under antibiotic stress by reducing metabolic activity.

The occurrence and isolation of *A. baumannii* with different Tgc resistance levels during antibiotic treatment may not have been identified by chance. Although sputum samples could be contaminated with other microbes, we could not determine whether the multiple isolates from the same patient stemmed from one strain; the three isolates were phylogenetically associated with a rare strain type (ST), which increased the possibility that a bacterial “evolution” process occurred that was reflected in the surviving dominant strain during antibiotic treatment. The occurrence of intermediately resistant and resistant strains has been repeatedly observed in our studies, and it supports the fitness cost (e.g., slow growth) theory of bacteria^14^. It has been reported that, upon antibiotic withdrawal, the ancestral strain with reduced resistance may outcompete the resistant isolate^13^. It has been proven that the bacterial metabolism state is closely related to AR^23^,^24^. In our case, the resistant strain 2015ZJAB2 had reduced metabolic transcriptional activity compared to the intermediate strain 2015ZJAB1, as represented by amino acid biosynthesis and glycolysis pathways under the Tgc concentrations of 0.5 and 1 mg/L (Fig. 6). The reduced metabolism may help the resistant strain succeed in the Tgc environment, but the withdrawal of antibiotics might not favor its proliferation. Instead, the withdrawal of antibiotics could have facilitated 2015ZJAB3 three weeks after Tgc treatment.

In summary, this study has provided the first report of *in vivo* Tgc resistance development in clinical *A. baumannii* isolates via putative homologous recombination with further transcriptome analysis to reveal significant gene profiles and metabolism characteristics related to resistance. Mutated genes associated with increased Tgc resistance may include OprD, a sugar-type MFS protein, and a LuxR-type transcriptional regulator. Notably, under Tgc conditions, the benzoate degradation pathway was actively regulated, and the TCA pathway was significantly down regulated, which may reflect *A. baumannii*’s capacity in an environment with microcide and adaptation by reducing its metabolic activity.

## Materials and Methods

### Bacterial isolates and preparation for Illumina sequencing

All six clinical isolates of *A. baumannii* were from three different patients who were treated at the First Affiliated Hospital of Zhejiang University. Each strain was purified for single colonies on Müeller-Hinton agar plates, and six strains were categorized as Patient I, Patient II and Patient III according to their source. The sample type, isolation time and antibiotics used during patient treatment are listed in Table 1 and Figure S1. All isolates were identified using the VITEK2 automated instrument for ID/AST testing (Bio-Mérieux, France) and a MALDI-TOF mass spectrometer (Bruker Microflex, Germany). The minimal inhibition concentration (MIC) of the clinical strains was determined using micro-dilution methods following guidelines by CLSI^25^. For all isolates, DNA was extracted using cultured cells in Müeller-Hinton broth collected at log phase and analyzed using a QiaAmp DNA mini kit according to the manufacturer’s instructions. MLST was performed based on Oxford methodology and seven housekeeping genes, *gltA, gyrB, gdhB, recA, cpn60, gpi* and *rpoD*^26^. Total RNA was isolated using the Ambion RNA kit from cultures of 2015ZJAB1, 2015ZJAB2 and 2015ZJAB3. Cultures were supplemented with 0, 0.5 and 1 mg/L Tgc (Sigma) and incubated for 13, 15 and 22 hours, respectively, to an optical density of approximately 0.8 at a 600-nm wavelength. DNA was then prepared for genome sequencing, and mRNA was purified for strand-specific transcriptome library construction according to the Illumina protocol.

### Genome sequencing, assembly, annotation and comparative genomics analysis of the isolated *A. baumannii*

Illumina sequencing, *de novo* assembly and functional annotation were performed by the Beijing Genomics Institute (Shenzhen, China). Illumina sequencing was performed using a HiSeq™ 2000 (Illumina, USA) in the paired-end mode with a read length of 100 bp. Briefly, DNA libraries for the 6 isolates were sequenced separately. After performing quality control on raw data with the NGS QC toolkit package (version 2.3), the acquired clean reads were then assembled by SOAPdenovo2. The parameters were optimized for best assembly contiguity statistics and the placement of a subset of high quality read pairs in the assembly with correct spacing, orientation, and comparisons to reference genome sequences^27^. The assembled genome sequence was annotated by Glimme V3.02^28^ for the identification of protein-coding genes, and rRNA and tRNA sequences were predicted by RNAmmerV1.2 and tRNAscan-SE, respectively^29^,^30^. To obtain functional annotation, the amino acid sequences of the predicted CDS were blasted against protein databases, including the NCBI nr database, COG, KEGG and Swiss-Prot databases, with parameters “-e 1e-5 -F F” for best hits^31^,^32^,^33^. ISs were identified using the IS Finder database (www-is.biotoul.fr)^34^.

To obtain drug resistance gene annotation, amino acid sequences of predicted genes were aligned against ARDB and CARD using blast^35^. A protein was assigned to an AR protein by the highest scoring annotated hit containing an identity of 40%.

Virulence annotation was performed using the VFDB database using blast (parameters: “-e 1e-5 -F F”)^36^.

### Comparative genomics analysis

The data used in comparative analysis were downloaded from the NCBI database (ftp://ftp.ncbi.nlm.nih.gov/GenBank/genomes/Bacteria/), including complete genome sequences and annotation of *A. baumannii* isolates BJAB07104 (GenBank accession no. CP000521). TRFV4.04 and RepeatMaskerV3.29 were employed to mask genome repeat regions, and then mummerV3.22 was employed to call SNPs. SNPs were confirmed by align reads back to the reference genome and filtered by the following cutoff values: average quality >20, not at the 5-bp edge of reads, and at least 10 high quality and good position support for SNP^37^. In the present study, SNPs were analyzed in Patient I strains with 2015ZJAB1 as the reference. Insertion and deletion analyses were carried out by alignment with the reference genome BJAB07104. Comparison of the isolates and reference genome BJAB07104 was performed using MUMmer^37^ and LASTZ^36^,^38^.

The value of Ka/Ks was determined by the Ka and Ks Calculator^39^. The phylogenetic tree was constructed with the array of core gene SNPs by TreeBestV1.9.2^40^ using the method of PhyML, and the setting of bootstraps was 1,000.

### Comparative transcriptome profiling of the three *A. baumannii* in Patient I

Transcriptome sequencing of the three *A. baumannii* in Group 1 (Patient I) under different Tgc concentrations was performed using HiSeq™ 2000 (Illumina, USA) at the Beijing Genomics Institute (Shenzhen, China). Raw reads were quality controlled using the following criteria: reads with an adaptor, with more than 2% unknown nucleotides, or with more than 20% low-quality bases (base quality ≤ 20) were removed. Next, the acquired clean data were mapped to the reference genome BJAB07104 with the Bowtie v2.2.5^41^. Reads Per Kilobase per Million mapped reads (RPKM), which considers the effect of sequencing depth and gene length for the reads, was used as a normalization step to quantify the gene expression levels^42^. Genes with an adjusted p-value < 0.05, FDR ≤0.001 and absolute value of log2 (treatment/control) ≥1 were identified as being differentially expressed^43^. Functional classification of differentially transcribed genes were annotated using the GO and KEGG databases. GO terms with a corrected p-value less than 0.05 were considered to be significantly enriched by differentially expressed genes.

### Validation of differentially expressed genes by real-time RT-PCR

To evaluate the reliability of our differentially expressed genes, primer pairs specific to 24 selected transcripts, including SNPs, Indels found in the genome and several genes with a 32-fold change with the increasing Tgc concentration, were designed for qRT-PCR. The 16S rRNA transcript was used as an internal standard within each sample. The relative expression levels of selected genes were calculated using the 2^−ΔΔCT^ method^44^. All gene specific primer sequences and gene IDs are listed in Table S6. Additionally, the PCR procedure was the same as described in our previous study^45^.

## Additional Information

**Accession codes:** The sequence data in this study has been deposited in NCBI under BioProject number PRJNA307212 for the genome sequences of the six *A. baumannii* strains, and PRJNA307155 for the seven strand-specific transcriptome sequences. 

**How to cite this article**: Liu, L. *et al.* Analysis of tigecycline resistance development in clinical *Acinetobacter baumannii* isolates through a combined genomic and transcriptomic approach. *Sci. Rep.* **6**, 26930; doi: 10.1038/srep26930 (2016).

## Supplementary Material

Supplementary Figure 1

Supplementary Table S1

Supplementary Table S2

Supplementary Table S3

Supplementary Table S4

Supplementary Table S5

Supplementary Table S6

Supplementary Table S7

Supplementary Table S8

## Figures and Tables

**Table 1:** Susceptibility profiles of six MDR *A. baumannii* strains isolated from three different patients.

Antibiotics	Group 1 (Patient I)			Group 2 (Patient II)	Group 3 (Patient III)	
	2015ZJAB1	2015ZJAB2	2015ZJAB3	2015ZJAB4	2015ZJAB7	2015ZJAB8
	MIC (mg/L)	MIC (mg/L)	MIC (mg/L)	MIC (mg/L)	MIC (mg/L)	MIC (mg/L)
Amikacin	256	256	64	64	64	128
Aztreonam	32	32	32	64	64	64
Cefepime	256	256	64	256	256	128
Ceftazidime	64	32	32	64	64	128
Imipenem	128	128	128	64	64	128
Levofloxacin	2	2	2	2	2	2
Piperacillin/Tazobactam	4	4	4	2	2	2
Tigecycline	4	8	6	4	4	8
Cefoperazone/Sulbactam	64	32	64	32	32	64

Note: The MIC of major clinical antibiotics and mixed drugs (cefoperazone/sulbactam = 1/1, piperacillin/tazobactam = 8/1) was determined by the microdilution method with fresh MHB medium according to the CLSI guidelines.

**Table 2:** General features of six *A. baumannii* genomes.

Characteristic	Group 1 (Patient I)			Group 2 (Patient II)	Group 3 (Patient III)	
	2015ZJAB1	2015ZJAB2	2015ZJAB3	2015ZJAB4	2015ZJAB7	2015ZJAB8
Source	sputum	sputum	sputum	bile	drain fluid	drain fluid
date to collect	2014 Jan 13	2014 Jan 28	2014 Feb 21	2012 Sep 22	2013 Jan 4	2013 Jan 14
Clean reads No.	7269296	7462466	7420560	8022386	6823616	7751334
Reads length/bp	90	90	90	90	90	90
Clean bases/bp	654236640	671621940	667850400	722014740	614125440	697620060
Genome size/bp	3954957	3964372	3943968	3942129	4101209	4076260
N50/bp	177993	177950	177986	184628	176354	176352
N90/bp	50999	50405	72813	48099	41163	41159
Scaffold No.	56	53	41	53	67	62
Contig No.	59	58	45	57	76	66
GC content/%	38.91	38.92	38.99	38.82	39.05	39.02
CDS No.	3,747	3,753	3,708	3,709	3,909	3,881
tRNAs No.	68	67	64	67	70	68
rRNAs No.	4	4	3	4	4	3
