# Supplementary material for: Analysis of tigecycline resistance development in clinical Acinetobacter baumannii isolates through a combined genomic and transcriptomic approach
Source: Sci Rep. 2016 May 31;6:26930. doi: 10.1038/srep26930 (PMC4886253; doi:10.1038/srep26930)
Supplement: Supplementary Figure 1 [file srep26930-s1.pdf]

Analysis of tigecycline resistance development in clinical *Acinectobacter baumannii* isolates through a combined genomic and transcriptomic approach

Lin Liu[1,2], Yujun Cui[3], Beiwen Zheng[1,2], Saiping Jiang[1,2], Wei Yu[1,2], Ping Shen[1,2], Jinru Ji[1,2], Lanjuan Li[1,2], Nan Qin[1,2]*, Yonghong Xiao[1,2]*

[1]State Key Laboratory for Diagnosis and Treatment of Infectious Disease, The First Affiliated Hospital, College of Medicine, Zhejiang University, 310003 Hangzhou, China

[2]Collaborative Innovation Center for Diagnosis and Treatment of Infectious Diseases, Zhejiang University, 310003 Hangzhou, China.

[3]State Key Laboratory of Pathogen and Biosecurity, Beijing Institute of Microbiology and Epidemiology, Beijing, China.

* Correspondence and requests for materials should be addressed to Yonghong Xiao (xiao-yonghong@163.com) and Nan Qin (qinnan001@126.com)

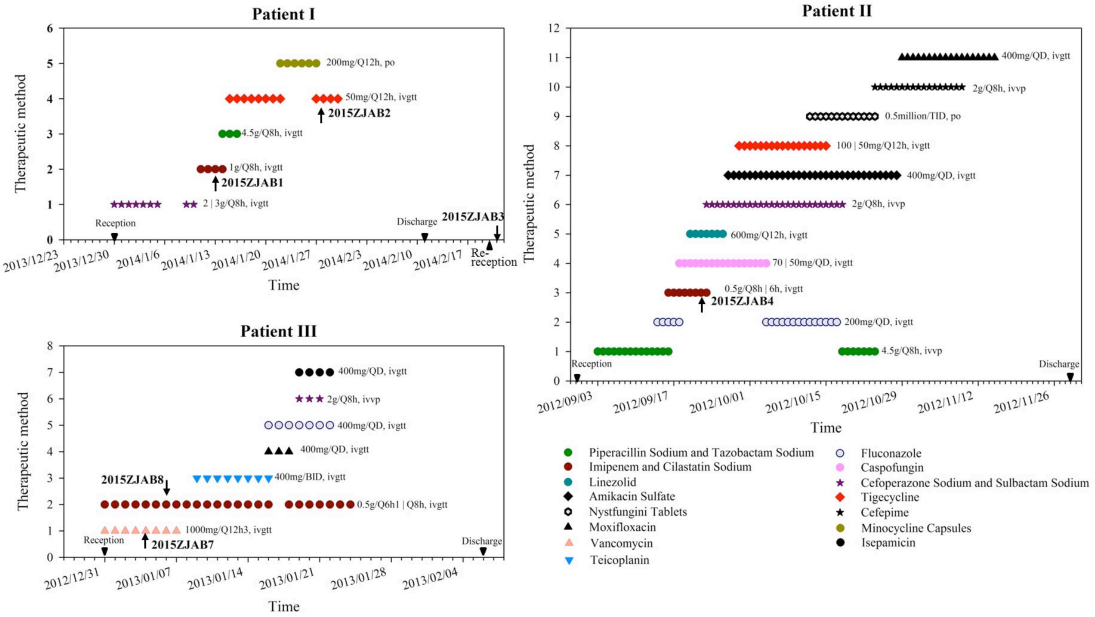

Figure S1. Clinical antibiotic treatment and isolation of the six *A. baumannii* strains in three patients. Symbols represent the different antibiotics and accompanying enzyme inhibitors used in clinical treatment, the black triangle indicates the reception time and discharge time of the patients, and the black arrows represent the time points when the clinical samples were taken and strains were isolated. Abbreviations: ivgtt for intravenous guttae, po for per os, ivvp for internal vertebral venous plexus, and QD for once per day, Q8 h for every 8 hours, Q12 h for every 12 hours. The vertical molding (|) indicates the different period of a therapeutic method used.
