# Supplementary material for: Analysis of tigecycline resistance development in clinical Acinetobacter baumannii isolates through a combined genomic and transcriptomic approach
Source: Sci Rep. 2016 May 31;6:26930. doi: 10.1038/srep26930 (PMC4886253; doi:10.1038/srep26930)
Supplement: Supplementary Table S3 [file srep26930-s4.pdf]

Analysis of tigecycline resistance development in clinical *Acinectobacter baumannii* isolates through a combined genomic and transcriptomic approach

Lin Liu[1,2], Yujun Cui[3], Beiwen Zheng[1,2], Saiping Jiang[1,2], Wei Yu[1,2], Ping Shen[1,2], Jinru Ji[1,2], Lanjuan Li[1,2], Nan Qin[1,2]*, Yonghong Xiao[1,2]*

[1]State Key Laboratory for Diagnosis and Treatment of Infectious Disease, The First Affiliated Hospital, College of Medicine, Zhejiang University, 310003 Hangzhou, China

[2]Collaborative Innovation Center for Diagnosis and Treatment of Infectious Diseases, Zhejiang University, 310003 Hangzhou, China.

[3]State Key Laboratory of Pathogen and Biosecurity, Beijing Institute of Microbiology and Epidemiology, Beijing, China.

* Correspondence and requests for materials should be addressed to Yonghong Xiao (xiao-yonghong@163.com) and Nan Qin (qinnan001@126.com)

Supplemental materials

Table 3. IS sequences in group I *A.baumannii* strains.

qurey		target	Identity	align_len	mismatch	gap	q.stat	q.end	t.stat	t.end	e-value	score	**t.length**
2015ZJAB1	scaffold5	ISAba22	100	1274	0	0	264743	266016	1	1274	0	2526	**1274**
	scaffold13	ISVsa3	100	977	0	0	43445	44421	977	1	0	1937	**977**
	Contig7	ISEc29	100	1325	0	0	4775	6099	1	1325	0	2627	**1325**
	Contig7	ISEc28	99.78	897	2	0	2226	3122	1	897	0	1762	**897**
	Contig7	ISEc35	84.97	825	124	0	2226	3050	155	979	0	652	**1051**
	Contig19	ISAba1	100	1124	0	0	1	1124	1137	14	0	2228	**1180**
2015ZJAB2	scaffold5	ISAba22	100	1274	0	0	264721	265994	1	1274	0	2526	**1274**
	scaffold14	ISVsa3	100	977	0	0	43423	44399	977	1	0	1937	**977**
	Contig6	ISEc29	100	1325	0	0	6968	8292	1325	1	0	2627	**1325**
	Contig6	ISEc28	99.78	897	2	0	9945	10841	897	1	0	1762	**897**
	Contig6	ISEc35	84.97	825	124	0	10017	10841	979	155	0	652	**1051**
	Contig16	ISAba1	100	1124	0	0	1	1124	14	1137	0	2228	**1180**
2015ZJAB3	scaffold13	**ISVsa3**	100	977	0	0	61990	62966	1	977	0	1937	**977**
	Contig14	ISAba19	100	1309	0	0	1	1309	1309	1	0	2595	**1309**
	Contig14	ISAba2	91.68	1310	106	3	1	1309	1308	1	0	1709	**1308**
	Contig14	ISAba18	88.08	1309	156	0	1	1309	1309	1	0	1358	**1309**
	Contig16	**ISAba1**	100	1124	0	0	1	1124	1137	14	0	2228	**1180**
